# Using transfer learning from prior reference knowledge to improve the clustering of single-cell RNA-Seq data

**DOI:** 10.1038/s41598-019-56911-z

**Published:** 2019-12-30

**Authors:** Bettina Mieth, James R. F. Hockley, Nico Görnitz, Marina M.-C. Vidovic, Klaus-Robert Müller, Alex Gutteridge, Daniel Ziemek

**Affiliations:** 1 Machine Learning Group, Technische Universität Berlin, Berlin, 10587 Germany; 2 Department of Pharmacology, University of Cambridge, Cambridge, CB2 1PD United Kingdom; 3 GlaxoSmithKline, Stevenage, SG1 2NY United Kingdom; 4 Department of Brain and Cognitive Engineering, Korea University, Seoul, 02841 Republic of Korea; 5 Max Planck Institute for Informatics, Saarbrücken, 66123 Germany; 6 Pfizer, Worldwide Research and Development, Berlin, 10785 Germany

**Keywords:** Functional clustering, Machine learning, RNA sequencing

## Abstract

In many research areas scientists are interested in clustering objects within small datasets while making use of prior knowledge from large reference datasets. We propose a method to apply the machine learning concept of transfer learning to unsupervised clustering problems and show its effectiveness in the field of single-cell RNA sequencing (scRNA-Seq). The goal of scRNA-Seq experiments is often the definition and cataloguing of cell types from the transcriptional output of individual cells. To improve the clustering of small disease- or tissue-specific datasets, for which the identification of rare cell types is often problematic, we propose a transfer learning method to utilize large and well-annotated reference datasets, such as those produced by the Human Cell Atlas. Our approach modifies the dataset of interest while incorporating key information from the larger reference dataset via Non-negative Matrix Factorization (NMF). The modified dataset is subsequently provided to a clustering algorithm. We empirically evaluate the benefits of our approach on simulated scRNA-Seq data as well as on publicly available datasets. Finally, we present results for the analysis of a recently published small dataset and find improved clustering when transferring knowledge from a large reference dataset. Implementations of the method are available at https://github.com/nicococo/scRNA.

## Introduction

**Figure 1:**
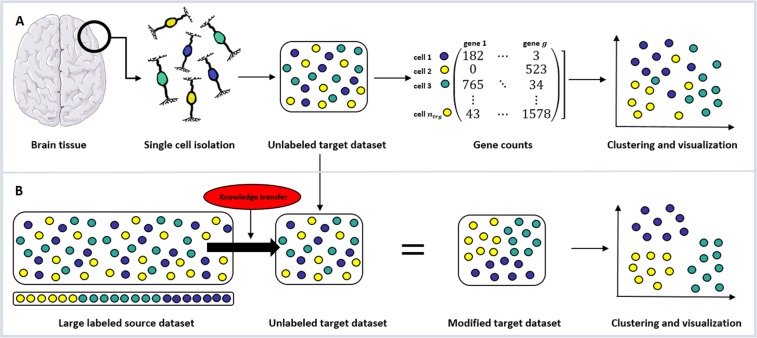
Single-cell RNA sequencing (scRNA-Seq) and transfer learning. (**A**) Recent scientific and biotechnological developments have enabled scRNA-Seq, the accurate measurement of the transcriptional output of individual cells. Once a tissue sample (e.g. brain tissue) is extracted from an organism, single cells (e.g. neurons) are isolated and sequenced. For each gene the number of times a corresponding transcript is found in each individual cell is counted. These gene expression profiles of single cells are then used to identify tissue-specific cell types or states through an unsupervised clustering algorithm (e.g. SC3) which can eventually be visualized (through e.g. t-SNE or PCA plots). (**B**) When clustering smaller disease or tissue specific scRNA-Seq datasets it is often desirable to utilize large labeled reference datasets. The current work proposes to apply the machine learning concept of transfer learning to modify the unlabeled target dataset via Non-negative Matrix Factorization (NMF) in a way that reflects specific properties of a large labeled source dataset and improves the results of downstream clustering algorithms (in our case SC3). Please note, that even though this graph represents a complete overlap in cell types, both source and target datasets might include cell types that are not part of the other set. Graphs were created using Servier Medical Art (brain, neuron and syringe) according to a Creative Commons Attribution 3.0 Unported License guidelines 3.0 (https://creativecommons.org/licenses/by/3.0/). Colour changes were made to the original neuron cartoons.

Sorting objects into groups with limited, or no, *a priori* knowledge, is a common problem in many different areas of scientific research^1,2^. In biological and medical sciences, datasets are often constrained by the scarcity, feasibility and expense of collecting samples. As such, it is not straightforward to apply state-of-the-art methodologies, like deep learning, which require large and well annotated datasets to many problems. To address this, the concept of using transfer learning to integrate *a priori* knowledge from large reference datasets into smaller datasets has been proposed as one way to generate additional insights from these data^3,4^. One of the scientific fields where these problems are of interest is single-cell RNA sequencing (scRNA-Seq). Figure 1 shows a graphical representation of the scRNA-Seq procedure and the application of transfer learning to its specific problem setting.

In recent years, a series of advances in molecular biology^5–7^, microfluidics^8,9^ and data analysis^10^ have led to our ability to accurately measure the transcriptional output of large numbers of individual cells through scRNA-Seq (Fig. 1A). The application of this technology has already led to insights into cellular development^7,11^, dynamics^12^ and heterogeneity^13,14^ and the pathogenesis of human disease^15^. The advent of major global initiatives focusing on scRNA-Seq such as the Human Cell Atlas^16^ means that the importance and impact of this technology is likely to grow as will the associated data analysis challenges.

Most scRNA-Seq experiments are concerned with the identification and cataloging of cell types or states within a tissue or biofluid^17,18^ (Fig. 1A). Historically this has been done through measurement, often qualitatively, of small numbers of ‘marker’ genes whose expression has been observed to correlate with cellular function. scRNA-Seq complements these approaches by being high-throughput, quantitative and cost effective in generating high dimensional data suitable for cell type classification. Neuronal cell types, for instance, have been deeply studied by scRNA-Seq^19^ leading to new, unbiased, data driven classifications of neurons and other cell types within the mammalian peripheral and central nervous systems^20–26^. Unique disease-associated cell states such as microglial subtypes associated with Alzheimer’s Disease^15^ have also been identified by scRNA-Seq.

Whilst analysis of scRNA-Seq data has many challenges including normalization^27,28^, noise^29^, dealing with zero inflation and missing values^30,31^, dimensionality reduction^30,32^ and visualization^10,33^, one of the key analytical techniques to address questions of cell type identification is that of unsupervised clustering. Clustering of cells into discrete groupings according to their transcriptional state is the fundamental analysis required in many scRNA-Seq experiments.

A range of approaches have been taken to address the problem of clustering scRNA-Seq data including hierarchical and iterative clustering^34,35^, PCA based approaches^20,36^, ensemble clustering^35,37^ and graph based approaches^38–42^. As the number of cells in scRNA-Seq datasets increases, the development of other machine learning based^43,44^, and specifically deep learning-based^45–48^, clustering approaches has expanded.

Challenges remain in the field, especially when the number of cells profiled in a given experiment is relatively small and as such rare cell subtypes are poorly represented^39^. Our hypothesis is that large reference scRNA-Seq datasets are a hitherto untapped resource for clustering of other datasets that may be smaller in size, but examine a specific tissue or disease context. Here we propose that the concept of transfer learning (the machine learning concept of applying knowledge gained from one context to another distinct but related context) can be effectively implemented to improve clustering of scRNA-Seq data when a suitable reference dataset is available (Fig. 1B).

Transfer learning is an umbrella term for problems such as multitask learning, domain adaptation, and covariate shift^3^. Specifically, it refers to a setting where the solution of one or multiple source tasks is applied to a related target task. Thrun^49^ contributed to the emergence of the field by asking if “Learning The n-th Thing [is] Any Easier Than Learning The First?” which was motivated by findings in human psychology. One of the key insights was that humans build upon related concepts when learning new tasks, which Thrun coined *lifelong learning*. Another influential line which popped up around the same time, introduced the term multitask learning^50^. Instead of learning a sequence of related tasks, multiple related tasks are learned in a parallel fashion using a shared representation.

In the analysis of scRNA-Seq data this translates to a situation where we are interested in simultaneously clustering a number of different datasets stemming from different studies, laboratories or points in time. These kinds of datasets most likely contain batch effects which need to be corrected for when combining the datasets for meta-analyses. In scRNA-Seq analysis, clustering and batch effect removal are typically addressed through separate steps, *i.e*. only after removing batch effects and combining multiple datasets into one is clustering analysis performed. These kinds of batch effect correction approaches can be graph-based^51–55^, dimensionality-reduction based and variance-driven^56–58^ or incorporate deep-learning procedures^59,60^. Different approaches to grouping cells of multiple datasets by cell type rather than dataset-specific conditions put emphasis on performing batch effect removal jointly with the clustering analysis^61–63^. More general approaches compare subtypes of cells across different samples^64^ and identify clusters with high similarity across datasets^65,66^.

All of the aforementioned methods presume that the multiple datasets under investigation are related in some way and are subsequently clustered simultaneously. In this work we focus on a more specific problem setting, where the user is interested mostly in the clustering of a target dataset, making use of the knowledge from a well-known and well understood source dataset. A number of tools are available for annotating cells of a target dataset to a predefined reference set of cell types^67–70^, but they are limited to target datasets that only include cells of the same types in source data and hence, cannot identify new cell types.

To enable knowledge transfer without having to combine the two datasets and at the same time guarantee a target clustering to be independent of the cell types of the source data, this work focuses on the specific concept of transfer learning to use information from one scRNA-Seq dataset to annotate another without limiting the cell types that may be found in either. The aim is to adjust the target dataset with information from the source data and feed this new target dataset into a downstream clustering algorithm.

In this specific setting one method that is the most closely related to our work is SAVER-X^71^. SAVER-X trains a deep autoencoder on a target set with an initialization of the weights obtained from training on source dataset coupled to a Bayesian model to leverage existing data in the denoising of a new scRNA-Seq dataset. SAVER-X is a deep-learning based approach and is thus limited to datasets of very large sample size. Our work focuses on improving the clustering of small datasets and does not require large sample sizes. Unlike deep learning based approaches, our method is convex and always returns the globally optimal solution independent of the initialization. Additionally, instead of focusing on denoising target datasets like SAVER-X we are trying to insert additional knowledge (*i.e*. to induce certain specific properties of the source dataset that the researcher wants to put special emphasis on) into the target dataset. This is achieved by making use of specific source datasets and in particular by including cell type annotations from the source into the analysis. Large reference datasets are often very well studied and come with high quality annotation of the cell types present within them. Our algorithm is not attempting to re-cluster this already well-clustered data but it is making use of those pre-existing source labels (Fig. 1B).

Another relevant work focusing on transformations of scRNA-Seq data for improved cell type clustering^72^ is also deep-learning based and consists of three subsequent steps. Firstly, a supervised neural network is trained to predict the cell types of a given source dataset. Secondly, the target dataset of cell types not used in the training is plugged into the network and the values of the hidden layer are used as a new representation of the target dataset. Lastly, the newly constructed target dataset is clustered with unsupervised k-means clustering enabling cell types in source and target data to not be identical. Please note, that the focus of the present work lies on transferring knowledge between source and target datasets that have a significant overlap in their cell types. The method proposed by Lin *et al*.^72^ is explicitly restricted to non-overlapping settings.

To summarize, the current approach is not directly comparable to the methods presented here, because it tackles a very specific problem that - to the best of our knowledge - no other method has addressed.

Implementations of the method are available as a Python framework at https://github.com/nicococo/scRNA.

## Methods

We propose a method to apply transfer learning to scRNA-Seq data that enables us to transfer knowledge from a relatively well-annotated and large source dataset to a smaller unannotated target dataset. A graphical representation of the method can be found in Fig. 1B. The method is based on a transfer learning step, that modifies the target dataset to incorporate knowledge gained from the well-annotated source dataset. The newly constructed target dataset can then be analyzed with a clustering algorithm to obtain an improved clustering compared to applying that same method to the target without any transfer learning procedure or a simple concatenation of source and target.

The following sections describe the method in more detail and specify the experimental setup of performance assessments on generated synthetic data, controlled real data and a real-world application of two independent datasets.

### Transfer learning for scRNA-seq clustering

There exists a well-known source dataset *X*_*src*_ with scRNA-Seq data from *n*_*src*_ cells and *g* genes for which we have in-depth knowledge about the clustering structure (i.e. ground truth labels ${y}^{src}\in {\Re }^{{n}_{src}}$) and a target dataset *X*_*trg*_ of *n*_*trg*_ cells and *g* genes which we want to enhance given the information in *X*_*src*_ and *y*^*src*^ before clustering into *k* groups of cells.

The basic underlying idea of the proposed method is to factorize the source dataset into a gene independent part (of size *k* × *n*_*src*_) and a data size independent part (of size g × *k*) and to use the latter – which is often called a *dictionary* since it does not depend on the number of cells *n*_*src*_ and can thus be used to *translate* between datasets – to modify the target dataset accordingly.

More specifically, the novel approach, based on non-negative matrix factorization (NMF), can be derived in the following steps:

1. We use NMF^73,74^ of our source data ${X}_{src}\in {\Re }^{g\times {n}_{src}}$ to learn a dictionary ${H}_{src}\in {\Re }^{g\times k}$ and a data matrix ${W}_{src}\in {\Re }^{k\times {n}_{src}}$ while regularizing the denseness of the results with an elastic net^75^:
  $$\begin{array}{rcl}{H}_{src},{W}_{src} & = & argmi{n}_{W,H}(\frac{1}{2}{||{X}_{src}-HW||}_{Fro}^{2}+\alpha \lambda ({||vec(H)||}_{1}+{||vec(W)||}_{1})\\  &  & +\,\frac{\alpha }{2}(1-\lambda )({||H||}_{Fro}^{2}+{||W||}_{Fro}^{2}))\end{array}$$
  Here, *λ* is the elastic net mixing parameter controlling the combination of L1 and L2 regularization and *α* is the corresponding penalty multiplier.
  As an initial starting point ${W}_{src}^{\ast }$ for *W*_*src*_ we provide a one-hot-encoding of the given cluster labels *y*^*src*^, where a non-zero entry in the *j*-th row of column *i* in ${W}_{src}^{\ast }$ indicates that cell *i* is a member of cluster *j*.
2. Given the learned dictionary ${H}_{src}\in {\Re }^{g\times k}$ from step (1) and assuming the genes in source and target data correspond, we now transfer knowledge from the source to the target dataset through the dictionary by learning a target data matrix ${W}_{trg}\in {\Re }^{k\times {n}_{trg}}$:
  $${W}_{trg}=argmi{n}_{W}(\frac{1}{2}{||{X}_{trg}-{H}_{src}W||}_{Fro}^{2})$$
3. To enable domain adaptation for different levels of cell type overlap between the two datasets we now construct a new target dataset ${X}_{trg}^{new}$ based on a convex combination of a reconstructed target dataset ${H}_{src}{W}_{trg}^{\text{'}}$ and its original version *X*_*trg*_:
  $${X}_{trg}^{new}=\theta {H}_{src}{W}_{trg}^{\text{'}}+(1-\theta ){X}_{trg}\,{\rm{with}}\,0\le \theta \le 1$$
  *θ* is a mixture parameter indicating how strongly knowledge from the source dataset should be transferred into the newly constructed target dataset. High values of *θ* indicate a strong influence of the source dataset on the modified dataset and low values cause the new dataset to be more similar to its original version.
  The target clustering matrix ${W}_{trg}^{\text{'}}\in {\{0,1\}}^{k\times {n}_{trg}}$ is a simplified version of *W*_*trg*_ with ones at the positions of all column-wise maxima and zeros elsewhere, *i.e*.
  $${({W}_{trg}^{\text{'}})}_{li}={1}_{[(argma{x}_{l\in \{1,\ldots ,k\}}{({W}_{trg})}_{li})=l]}\forall i,l$$
  Using ${W}_{trg}^{\text{'}}$ instead of *W*_*trg*_ represents reducing the information in this matrix to potential cluster memberships of the target cells which is appropriate considering the task at hand. To this end, a number of different approaches were implemented (e.g. leaving *W*_*trg*_ as it is or optimizing it in an additional training step), but it was found that taking the simplified version as described above performed best and most consistently for all scenarios under investigation.
4. The newly derived dataset ${X}_{trg}^{new}$ can be used as input for a clustering method. We are using single-cell consensus clustering (SC3)^37^ as an exemplary clustering method that is commonly used to solve scRNA-Seq clustering problems. See Supplementary Section 1.1. for a detailed description of SC3.

Please note that the proposed method does not inherently depend on the number of samples in each dataset and can technically (even though not studied in this work) be used to transfer knowledge from datasets of any size, not just from a source that is larger than the target.

### Selecting *θ* and other free parameters

The mixture parameter *θ* dictates how much the newly constructed target dataset should be changed by the information in the source dataset. *θ* is automatically chosen via an unsupervised assessment of the clustering quality through Kernel Target Alignment (KTA) scores^76^ which measure the similarity of kernels. The whole transfer learning and clustering procedure (steps 1–4) is applied with a number of values for *θ* within a pre-specified range and the KTA score between the linear kernel of the mixed dataset ${X}_{trg}^{new}$over the cells and the linear kernel of the cell type labels predicted by subsequent SC3 clustering is calculated. The parameter value yielding the optimal KTA score is chosen for the final result and can give an indication on the transferability between source and target data. An investigation of the mixture parameter of the transfer learning approach and its automatic selection process based on KTA scores is given in Supplementary Section 2.4. where the correlation of the unsupervised KTA scores and their supervised counterpart, the Adjusted Rand Indices (ARIs) are examined. Other free parameters, *e.g*. the elastic net parameters *λ* and *α*, were chosen based on results from the simulated data. See Supplementary Sections 2.2.,3.2. and 4.2. for details.

### Pre-processing

Three steps for pre-processing scRNA-Seq data were applied:

- **Cell filter:** Remove all cells containing fewer than *x*_*genes*_ genes with *expression* > *x*_*expression*_.
- **Gene filter:** Remove ubiquitous genes that are expressed in almost all cells (i.e. with *expression* > *x*_*expression*_ in at least *x*_*cells*_% of cells) and rare genes that are not expressed in almost all cells (i.e. with *expression* < *x*_*expression*_ in at least *x*_*cells*_% of cells).
- **Log-transformation:** Log-transform the expression matrix after adding a pseudo-count of 1.

All free pre-processing parameters should be selected by the user based on an inspection of the data, i.e. expression histograms of both source and target dataset. The specific parameter values chosen for the datasets in this work can be found in Supplementary Sections 2.1.,3.1. and 4.1. The corresponding expression histograms can be seen in Supplementary Figs. S5–S7. Pre-processing was performed once for all datasets (source and target separately) before the different clustering methods (i.e. transfer learning or baseline methods) were performed.

### NMF clustering in the absence of source labels

If no reliable cluster labels are available for the source dataset ${X}_{src}\in {\Re }^{g\times {n}_{src}}$, one can choose to generate those labels via NMF clustering^73,74^ and proceed as if they were the real labels *y*^*src*^. This basically consists of learning a dictionary ${H}_{src}\in {\Re }^{g\times k}$ and a data matrix ${W}_{src}\in {\Re }^{k\times {n}_{src}}$ as described above in step 1 and selecting the cluster memberships based on the column wise maxima of *W*_*src*_, i.e. ${y}_{i}^{src}=argma{x}_{l\in \{1,\ldots ,k\}}{({W}_{src})}_{li}$.

Instead of learning the source labels through NMF clustering one could also avoid providing an initial starting point ${W}_{src}^{\ast }$ for *W*_*src*_ when learning the dictionary *H*_*src*_ and the data matrix *W*_*src*_.

### Baseline methods and performance metrics

For assessing the quality of our unsupervised domain adaptation solution, we are interested in investigating the change of clustering accuracy of the target dataset. As baseline methods we implement the original SC3 clustering method on the target dataset alone (TargetCluster) and on the concatenated dataset of source and target (ConcatenateCluster). For a detailed description and a visualization of the baseline methods please see Supplementary Section 1.2 and Supplementary Fig. S2.

As a supervised performance metric we used the Adjusted Rand Index (ARI)^77^ comparing the transfer learning results (TransferCluster) and the baseline labels with the known clustering labels (known perfectly in the case of the simulated data and retrieved from the original publication in the case of the real data). ARI scores are computed only on the target data, even in the case of ConcatenateCluster, where labels are computed for both source and target cells.

### Simulation of source and target single-cell datasets

To test the applicability of our method we first use it on simulated count level scRNA-Seq data from a defined hierarchical set of clusters that represent the different cell types present in a tissue or biofluid.

**Figure 2:**
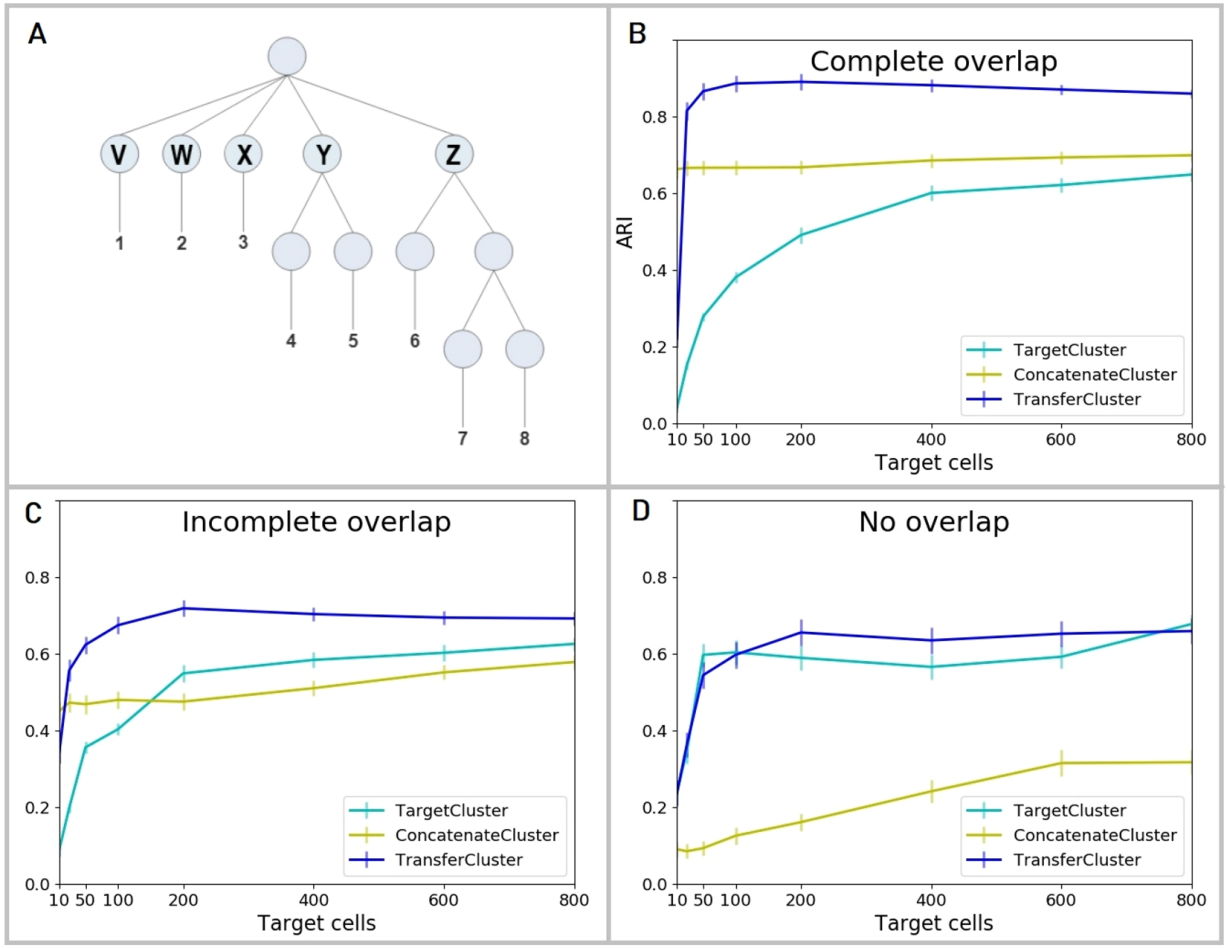
scRNA-Seq simulation data description and results. (**A**) Count level single-cell RNA-Seq data is simulated according to a pre-defined hierarchical clustering structure with eight cell clusters (1-8) that are derived from five top level clusters (V - Z). Generated datasets are individually split up by randomly assigning the top node clusters V - Z to source or target. Three different settings are considered: 1. both source and target data contain cells from all top node clusters V - Z (Complete overlap), 2. three randomly selected top node clusters V - Z are chosen as common to both source and target, the other two are assigned to either one of source and target (Incomplete overlap) or 3. cells from two of the top node clusters form the target dataset and cells from the other three form the source (No overlap). (**B)** Clustering performances of the baseline methods, TargetCluster (clustering on the target dataset alone) and ConcatenateCluster (concatenating and clustering source and target data simultaneously), and the transfer learning approach (TransferCluster) when the clustering structures of source and target data are identical (Complete overlap). (**C)** Clustering performances of the baseline methods, TargetCluster and ConcatenateCluster and the transfer learning approach (TransferCluster) for an incomplete overlap between the cell clusters in source and target data (Incomplete overlap). (**D)** Clustering performances of the baseline methods, TargetCluster and ConcatenateCluster and the transfer learning approach (TransferCluster) for a setting with two exclusive target and three exclusive source top nodes and no cell types that appear in both sets (No overlap). Please note, that due to the sampling procedures described above, the number of top level nodes in the target datasets decreases from 5 in (**B)** to 4 in (**C)** and 2 in (**D**) and hence the performance of TargetCluster improves from (**B–D**). 95% confidence intervals are shown.

Figure 2A shows a graphical representation of the hierarchical clustering structure used to generate the simulated data. Each generated dataset consisted of eight clusters of cells (1–8) deriving from five top level clusters (V - Z) that share a common background distribution of gene expression levels and some proportion of genes differentially expressed between them.

An outline of the data generation procedure is given here; the full code is provided in https://github.com/nicococo/scRNA/blob/master/scRNA/simulation.py. First we generate the number of cells in each sub-cluster using a Dirichlet distribution with a concentration parameter of 10. Then we define a common background distribution of gene expression levels sampled from a gamma distribution with shape 2 and rate 0.1. For each cluster in turn we randomly select 10–40 % of genes to be differentially expressed relative to the background. The difference in expression, expressed as a log2 fold change, for each such gene is sampled from a normal distribution with mean 1 and standard deviation 0.5. For clusters that are not themselves top level clusters (clusters 4–8) this process continues recursively with further expression differences generated for each sub-cluster using the parent cluster as the new background until the final clusters are reached. Finally, we generate count level data by applying a small amount of random normally distributed noise to the expression levels of each cell and then sampling the per gene counts from a negative binomial distribution with dispersion 0.1. The resulting datasets contain cells with a median count of 215 500 reads per cell. Please see Supplementary Section 2.3 and Supplementary Fig. S3 for details.

Once count level data is generated for the entire dataset we split it into target and source datasets with different sets of cells according to the cluster structure and the relationship between the target and source. Here, we consider three such relationships that reflect three possible experimental scenarios:

- Cells in the target and source are randomly sampled from the same underlying tissue or biofluid and hence contain cells from all top node clusters V–Z.
- Certain clusters are specified to be only present in the source and some to be only present in the target, the remaining clusters are present in both target and source. Three randomly selected top node clusters V - Z are chosen as common to both source and target, the other two are assigned to either one of source and target.
- The cells in the target and source are drawn from completely non-overlapping clusters. In this scenario transfer learning is not expected to be successful. Cells from two of the top node clusters form the target dataset and cells from the other three form the source.

The genes measured in source and target are always the same and the top nodes are randomly assigned to either source or target for each repetition of the data generation process. 100 sets of simulated data were generated for each of the three settings simulating the expression levels of 10 000 genes in 1800 cells. 1000 cells were assigned to the source dataset and the set of 800 target cells was downsampled (i.e. 10, 50, 100, 200, 400, 600, 800 target cells) to investigate the performance of the transfer learning approach and its corresponding baseline methods when applied to datasets of varying sizes.

### Subsampling source and target data from a single dataset

Following the analysis of simulated data, we subsequently examined a real scRNA-Seq dataset. By subsampling both source and target dataset from the same single original dataset, we create an environment where the potential benefit of transfer learning can be determined on real-world gene expression data. For this we utilized gene expression data provided as Reads Per Kilobase of transcript per Million mapped reads (RPKM) derived from over 1600 cells of the primary visual cortex of the adult mouse brain^26^. After the application of the pre-processing steps (see Supplementary Section 3.1. for details) the dataset contained expression levels of 9547 genes in 1658 cells.

We deemed this dataset to be of sufficient complexity in terms of taxonomic diversity (it contains 23 GABAergic neuronal, 19 glutamatergic neuronal and 7 non-neuronal cell types) and in terms of total cell count, to enable cluster-restricted subsampling and thus the application of transfer learning approaches.

We ran 100 repetitions splitting the data into a source dataset of 1000 cells and a target dataset of 650 cells each time, which is subsampled even further (to 25, 50, 100, 200, 400, 650 target cells) to assess performance for different sample sizes. To investigate the influence of complete and incomplete overlap between the clusters of source and target datasets, transcriptomic cell types assigned to either dataset were controlled. Complete overlap meant randomly assigning cells into source and target. Incomplete overlap is achieved by assigning the two largest clusters of the dataset (*Glutamatergic L4* cells and *GABAergic Pvalb* cells) to be either an exclusive source or an exclusive target cluster, respectively. All other clusters are shared amongst both source and target in this setting.

The transfer learning approach and its baselines are now investigated under two different conditions. Firstly, we assume that no ground truth labels are available and generate labels for 18 cell clusters via NMF clustering^73,74^ on the whole dataset. We interpret this clustering, based as it is on the totality of the data, as a ground truth clustering and apply our method and the baseline algorithms to a subset of the dataset, to see how each method performs relative to this definition of ground truth when not all of the data is available. Secondly, we use the data driven clustering labels provided in the original paper and take those as the ground truth labels. Specifically, we use a cut-off in the provided clustering hierarchy that results in 18 clusters. Given those alternative ground truth labels, we once again run TargetCluster, ConcatenateCluster and TransferCluster. See Supplementary Section 3.3. for a more detailed description of the two different sets of ground truth labels.

### Independent source and target dataset

As a real-world application of the transfer learning approach we analyze two entirely independent, but biologically related, datasets. To improve the clustering results of a relatively small target dataset from Hockley *et al*. 2018^21^, we transfer knowledge from a larger source dataset from Usoskin *et al*. 2014^20^ both derived from the rodent somatosensory system. The somatosensory system is responsible for detecting mechanical, thermal and chemical stimuli to which an organism can choose to elicit a behavioural response. Primary sensory neurons innervate the vast majority of internal hollow organs, joints, muscles and the skin evoking conscious sensation in the event of these stimuli. This is most clearly exemplified by pain in the case of potentially harmful or noxious stimuli, such as burning or cutting of the skin. In Usoskin *et al*, transcriptomic analysis of 622 primary sensory cell bodies, which reside within the dorsal root ganglia (DRG), reveals significant diversity in cell type (11 types) and sensitivity to a diverse range of stimuli modalities (e.g. thermosensitive, itch sensitive, nociceptive) to which an organism is exposed. However, previous retrograde tracing experiments show that only 5–10 % of DRG neurons project to internal (visceral) targets, such as the gastrointestinal tract, and as such are likely only represented by ~ 30–60 cells in the Usoskin *et al*. dataset. Such small cell numbers limit subtype assignment of cells in this organ. In order to overcome this limitation, scRNA-Seq has been performed on retrograde labeled DRG neurons known to selectively innervate the gastrointestinal tract (colonic DRG neurons), providing transcriptomic analysis of 314 cells from this specific organ that cluster into 7 distinct subtypes^21^. However, it is unclear whether *de novo* clustering of colonic DRG neurons identifies established clusters previously identified in larger datasets such as Usoskin *et al*. (hereafter designated ‘Usoskin’) or whether novel cell types exist within this dataset (hereafter designated ‘Hockley’).

After the application of cell and gene filter to the Hockley dataset provided as Transcripts Per Million (TPM), the number of genes decreased from 45513 to 9651; none of the 314 cells were filtered out. Pre-processing of the Usoskin dataset provided as Counts Per Million (CPM) left 9280 of the 20191 genes and 501 of the 622 cells. Please see Supplementary Section 4.1. for an investigation of the specific expression levels in these datasets and the parameter values that were consequently chosen for pre-processing. Since the methods require both source and target to have identical feature space, only the subset of genes that appear in both source and target data were used, leaving us with 4402 genes in both sets. In initial experiments, the original source and target data were used, however in later experiments, a batch effect removal approach was applied to control for the integration of single-cell transcriptomic data across different conditions and technologies. Here, we applied Seurat batch effect removal^56^ to combine the Hockley and the Usoskin data, and separated the result back into the original datasets which were then provided to TargetCluster, ConcatenateCluster and TransferCluster.

As an additional pre-processing step we investigated the effect of imputation on the clustering results. MAGIC^78^, a widely used method for imputing missing values to overcome zero-inflation in scRNA-Seq data, was applied to both datasets and the pre-processed datasets were then provided to the three methods under investigation.

Using either the original datasets or the pre-processed, batch effect removed or imputed datasets, the results of TargetCluster, ConcatenateCluster and TransferCluster were assessed in terms of performance via a comparison to the clustering of the original paper^21^, the evaluation of t-SNE plots and differentially expressed genes to determine putative cellular function to neuronal subtypes. Since SC3 is a non-convex method it yields different results for each run. In order to provide quantification of the robustness of the three methods, we applied each 1000 times and counted the number of times three key clusters of interest were successfully identified. These clusters were selected based on their biological relevance as described in the original paper, further details of which can be found in the results section.

Once again, experiments were run under two conditions. Firstly, we assumed that reliable source data labels are not available and we generated cell labels for the Usoskin dataset via NMF clustering. Secondly, we use labels from Usoskin *et al*. (generated via an iterative PCA approach). Usoskin *et al*. provide labels at three different levels of the hierarchy producing 4, 8 or 11 clusters. We investigate results based on all of those, calling them level 1, 2 and 3 labels, respectively. We also investigate a scenario where we generate the labels via NMF clustering instead of using the labels presented in Usoskin *et al*. Here, however, we only present results based on using level 3 labels from the original publication. Please see Supplementary Section 4.3 and 4.4 for a detailed description of the different sets of ground truth labels and the corresponding clustering results.

In order to assess whether rare cell types were present in the Hockley dataset, the number of clusters to group the cells of the target dataset in was chosen to be 7.

## Results

### Validation using simulated source and target data

To assess the performance of the proposed method in comparison to the two baseline methods in a controlled environment, we conducted a number of simulation experiments with generated data, where the “ground truth” of the clustering structure is controlled and known. This allowed us to compute supervised performance metrics for each method and make objective statements about which method performs best.

Figure 2B–D show the ARI curves of all three methods on these simulated scRNA-Seq datasets for the three different settings of overlap between source and target described above.

For complete overlap in the clustering structures of the two datasets, *i.e*. identically sampled data, our method, TransferCluster, outperforms the baseline methods for all sample sizes of the target dataset (Fig. 2B). It exceeds not only the clustering on the target dataset alone (TargetCluster) but also performs better than concatenating and clustering source and target data simultaneously (ConcatenateCluster). The latter can improve the clustering of the target dataset, but fails to achieve the same levels of performance as TransferCluster. The main reason for this is that instead of predicting the labels of the source dataset - like ConcatenateCluster - TransferCluster uses the true source labels and incorporates that knowledge into the clustering of the target dataset. This effect is very strong here since the true source labels are completely known for the generated datasets.

The ARI curves on simulated data with both overlapping and non-overlapping clusters in source and target data, show that in this case transferring knowledge can still help the analysis of the target dataset and that TransferCluster outperforms both baseline methods, however not by the same amount as when a complete overlap is present (Fig. 2C). Concatenating the two datasets (ConcatenateCluster) can lead to an increased performance for larger target sample sizes where clustering the target data alone (TargetCluster) is more successful. Only incorporating the source knowledge via our transfer learning procedure (TransferCluster) can consistently improve the clustering results for all sample sizes.

Specifically, one should note, that the performance, as measured by ARI, of ConcatenateCluster decreases when there is a non-perfect overlap (in comparison to a complete overlap) and is greatly impaired when there are no overlapping clusters in source and target data. Combining two sets into one is not to be preferred in those cases.

The ARI curves on disparate, non-overlapping clusters show that, as expected, transferring information from a source dataset that is unconnected to the target dataset cannot improve clustering significantly (i.e. confidence intervals of TargetCluster and TransferCluster overlap) and using SC3 on the target dataset alone (TargetCluster) is to be preferred (Fig. 2D). For two exclusive target and three exclusive source top nodes and no cell types that appear in both sets (No overlap), concatenating source and target into one dataset (ConcatenateCluster) has a negative effect on the clustering of the target cells and should be avoided. Importantly and in contrast to the ConcatenateCluster, the use of TransferCluster does not significantly reduce clustering performance compared to *de novo* clustering of the target data alone and can keep the levels of performance as high as not taking the source data into account at all, as the method can choose a low mixture parameter when there is no overlap. This can be seen in Supplementary Fig. S4 where the mixture parameter selection process of TransferCluster via KTA scores is investigated.

To conclude, the transfer learning approach outperforms both baseline methods and works as expected for simulated scRNA-Seq data. For a detailed description and analysis of the data simulation experiments, see Supplementary Section 2.

### Validation using subsampled source and target data from a single dataset

**Figure 3:**
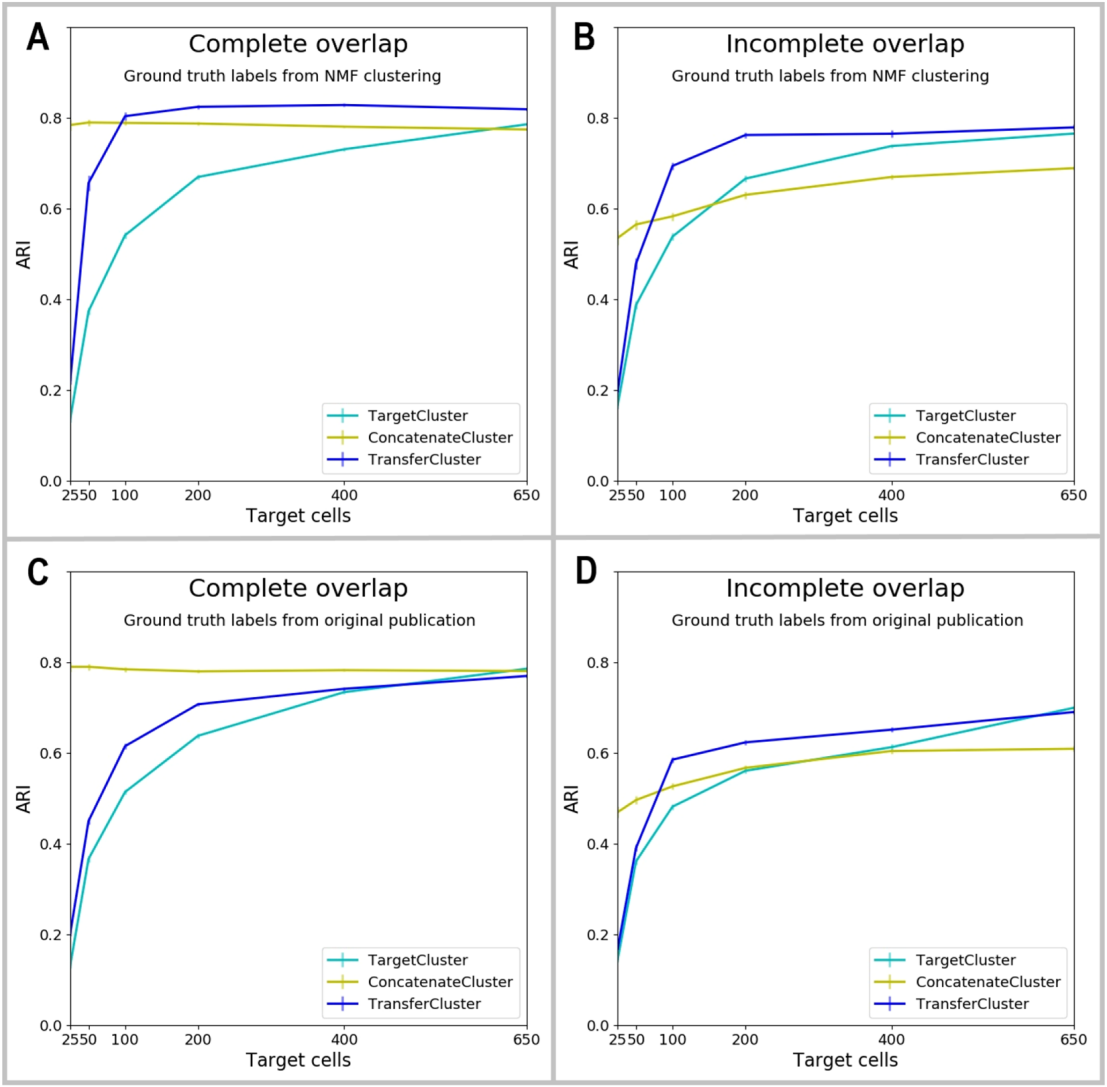
Results of source and target data from mouse visual cortex cells^26^. (**A**) Clustering performances of all three methods using NMF clustering labels of 18 clusters generated on the whole dataset as ground truth labels. Source and target datasets share the complete clustering structure, i.e. all cell types appear in both source and target data (Complete overlap). (**B**) Clustering performances of all three methods using NMF clustering labels of 18 clusters generated on the whole dataset as ground truth labels. The overlap is not complete, i.e. the two biggest clusters of the dataset are assigned to be either exclusive source or target clusters (Incomplete overlap). (**C**) Clustering performances of all three methods using the data driven clustering results from the original paper^26^ as ground truth labels. Source and target datasets share the complete clustering structure, i.e. all cell types appear in both source and target data (Complete overlap). (**D**) Clustering performances of all three methods using the data driven clustering results from the original paper^26^ as ground truth labels. The overlap is not complete, i.e. the two biggest clusters of the dataset are assigned to be either exclusive source or target clusters (Incomplete overlap). 95% confidence intervals are shown.

Now we present the results of subsampling both source and target from the same real scRNA-Seq dataset^26^ and comparing the performance of our method to that of the baseline methods. In order to validate our approach for a scenario where no reliable ground truth labels exist, we first generated synthetic labels of 18 clusters via NMF clustering^73,74^ on the whole dataset, which we then considered to be the ground truth for this experiment. Figure 3A,B show the corresponding ARI curves for complete and incomplete overlap between source and target dataset. For both scenarios, transferring knowledge into the target dataset improves its clustering in subsequent SC3 clustering and outperforms both baseline methods. When source and target datasets share the complete clustering structure (panel A), concatenating the two datasets (ConcatenateCluster) improves the clustering results of the target data (TargetCluster), but transferring knowledge via the proposed method (TransferCluster) is seen to improve it even more. While for a complete overlap, ConcatenateCluster can improve target clustering by a large margin, when the target dataset is relatively small in comparison to source (for example, 1/10th of source), the method fails to find additional gains over *de novo* clustering of target when the clustering structure in source and target are similar but not identical (Incomplete overlap, panel B). In this setting, which is the more realistic one in most cases, ConcatenateCluster does not perform well and only the knowledge transfer via the proposed method can improve the target clustering results. Hence, it should be the preferred option to incorporate source information into a target clustering.

Secondly, instead of generating labels of the complete dataset via NMF clustering, we use the data driven clustering labels provided in the original paper^26^ as ground truth labels and apply the same subsampling procedure as above. Figure 3C,D show the corresponding results for complete and incomplete overlap. Again, for both settings the transfer learning approach improves TargetCluster clustering on target data alone. Knowledge is successfully transferred from the source to the target dataset no matter how big the overlap in the clustering structure of the two sets is.

The comparison to the second baseline method of concatenating both sets into one, shows for complete overlap of the clusters in both datasets that transfer learning helps but cannot outperform ConcatenateCluster. However, in the more realistic setting of an incomplete overlap in the clustering structures, concatenating the two datasets has a negative effect on the target clustering, especially for large sample sizes. ConcatenateCluster collapses and it performs even worse than not using the source data at all (TargetCluster) for some larger target sample sizes. Transfer learning is able to avoid this effect and succeeds in incorporating valuable information from the source data into the target data improving its clustering results consistently for all target sample sizes. Transfer learning is clearly to be preferred in this setting.

### Biological results of independent source and target datasets

Leaving the controlled environment where source and target data are sampled from the same distribution, we lastly investigate a real-world application where source and target are completely independent, but biologically related, datasets, collected at different times and places. The proposed transfer learning method was able to identify relatedness but also differences in the two datasets by automatically choosing a mixture parameter θ of 0.7 in the KTA score procedure. Please see Supplementary Section 4.2 and Supplementary Fig. S8 where the mixture parameter selection process of TransferCluster via KTA scores is investigated.

**Figure 4:**
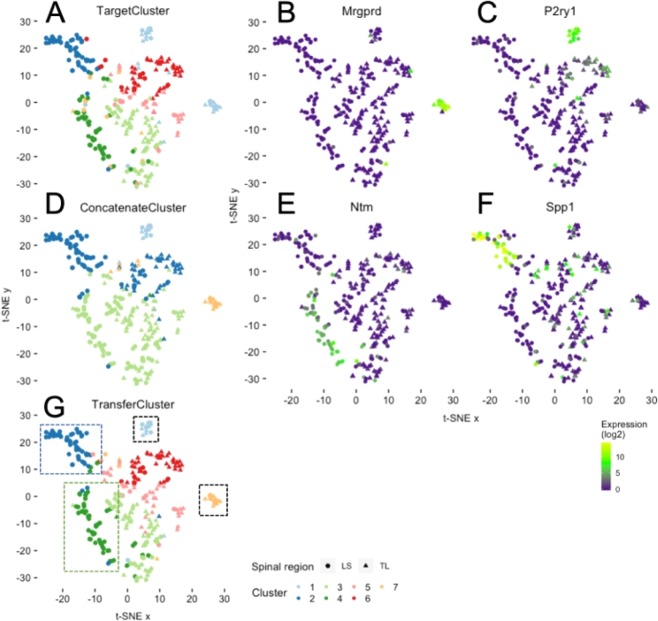
Clustering example biological data using the transfer learning approach. t-SNE plots of mouse colonic sensory neurons: colour refers to clusters derived from the three approaches (**A**) TargetCluster, using only data from Hockley *et al*.^21^ to assign clusters. (**D**) ConcatenateCluster, using a concatenation of data from Hockley *et al*.^21^ and Usoskin *et al*.^20^ (mouse sensory neurons) to assign clusters. (**G**) TransferCluster, using the novel transfer learning approach with Usoskin *et al*.^20^ as Source and Hockley *et al*.^21^ as Target) and shape refers to spinal segment from which the neuron was isolated (triangle, TL (thoracolumbar); circle, LS (lumbosacral)). In **G**, clusters 1 and 7 (black dashed boxes), cluster 2 (blue dashed box) and cluster 4 (green dashed box) represent biologically distinct groups of cells with differing sensory functions. This is exemplified by the cluster-specific expression of specific genes by cluster 7 (**B**), Mrgprd), 1 (**C**, P2ry1), 2 (**E**, Ntm) and 4 (**F**, Spp1). Colour scheme represents expression level [log(TPM)].

To assess the performance of our method, we are unable to compute ARI scores in this setting. In contrast to the simulations described above, the true underlying clustering architecture of the cells under study is largely unknown. Hence, we assessed clustering performance based on differential gene expression and biological relevance to known somatosensory pathways. In Fig. 4, we show t-SNE plots for the Hockley data overlaid with cluster memberships corresponding to the results of methods following the use of the Usoskin data as source. As predicted using TargetCluster (*i.e*. the method utilizing SC3 clustering of the Hockley data alone) we identified a similar cluster structure to that observed by the authors in their original study^21^. Specifically, we identify 6 well-defined clusters (and a 7th poorly defined cluster) that could be separated based on gene expression and also an important anatomical difference related to the spinal region from which the neuron was collected (*i.e*. in Fig. 4A, clusters 2 and 4 are both predominantly populated by lumbosacral sensory neurons as indicated by the use of circles, whilst the neurons within the other clusters are mainly thoracolumbar in origin as shown by triangles). In contrast to the original study, TargetCluster did however fail to robustly segregate two biologically distinct groups of cells, which, using the authors original nomenclature are named mNP and mNFa, respectively. In our hands, they correspond to cluster 1 in Fig. 4A. The first mNP cluster comprises 15 neurons and expresses Mas-related G-protein coupled receptor D (*Mrgprd*; Fig. 4B) and Lysophosphatidic acid receptor 3 (*Lpar3*); genes previously associated with non-peptidergic nociceptive pruriceptors^79^. The second mNFa group of 16 neurons expresses P2Y purinergic receptor 1 (*P2ry1*; Fig. 4C) and BAI1-associated protein 2-like 1 (*Baiap2l1*) and is indicative of mechanosensitive nociceptors^80^. Whilst SC3 was used by the authors to cluster in their original study, this is not deterministic and produces different results when solving the same clustering problem multiple times. Indeed, when we counted the number of times the mNP and mNFa clusters were separated when repeating the procedure, TargetCluster was only correct 224 times out of 1000. The use of ConcatenateCluster on both the Hockley and Usoskin datasets improved the robustness of clustering these two groups as separate clusters (506/1000, e.g. in Fig. 4D, clusters 1 and 7), however this came at the expense of clustering accuracy within the remaining neurons. For example, in Fig. 4D, ConcatenateCluster identifies a more simplistic cluster structure with 4 clusters and no longer distinguishes separations between spinal segmental regions (e.g. thoracolumbar and lumbosacral) from which neuronal subtypes have been collected. As such, the concatenation of target and source, in this instance at least, may miss biologically relevant clusters. Specifically, what the original authors suggest as a putative novel peptidergic subtype (pPEP) unique to the lumbosacral DRG with high expression of neurotrimin (*Ntm*; Fig. 4E), tyrosine hydroxylase (*Th*) and calcitonin polypeptide alpha (*Calca*), and a second group of lumbosacral neurons, the pNF subtype, which are thought to represent a low-threshold mechanoreceptor group within the colorectum with selective expression of secreted phosphoprotein 1 (*Spp1*; Fig. 4F) and the mechanotransducer *Piezo2*, would have been missed using ConcatenateCluster.

When knowledge from the larger Usoskin dataset was instead transferred using TransferCluster, not only was the clustering accuracy of the overall data retained (identifying 7 well-defined clusters) but the probability of separating the clusters mNP and mNFa was partially increased (for TransferCluster with level 3 labels, 352/1000; Fig. 4G). Unlike ConcatenateCluster, TransferCluster correctly identifies not only mNP and mNFa clusters (as highlighted by the black dashed boxes around clusters 1 and 7 in Fig. 4G) but also spinal region dependent clusters pPEP (green dashed box, cluster 4, Fig. 4G) and pNF (blue dashed box, cluster 2, Fig. 4G). In order to quantify these effects, we measured how frequently TransferCluster separated cluster 2 (e.g. pNF) from cluster 6 (1000/1000) compared to ConcatenateCluster (481/1000), and likewise, how frequently cluster 4 was separated from cluster 3 (887/1000) compared to ConcatenateCluster (4/1000). This clustering robustness analysis is summarized in Table 1.

**Table 1:** Robustness analysis on Hockley dataset.

	TargetCluster	ConcatenateCluster	TransferCluster
mNP/mNFa	224|230|479	506|998|902	352|605|919
pPEP	984|1000|921	4|33|431	887|1000|944
pNF	999|1000|801	481|962|579	1000|1000|831

For each method we present the number of times a specific cell type was identified correctly out of 1000 replications. In each field of the table the first number corresponds to applying the method to the original datasets with no additional pre-processing, the second number is the result of applying Seurat batch effect removal^56^ before the analysis and the third number represents results on datasets that have been imputed with MAGIC^78^.

In additional experiments, we applied an established batch effect removal pre-processing step^56^ to combine the Usoskin and Hockley datasets, which were then separated and our three clustering methods applied as described above. Batch effect removal improves the performances of both ConcatenateCluster and TransferCluster, however transfer learning still outperforms simultaneous clustering on the combined dataset. For example, ConcatenateCluster fails to reliably identify pPEP cells (33/1000), whilst TransferCluster following batch effect pre-processing finds all three cell types of interest in the majority of cases (mNP/mNFa split: 605, pPEP: 1000 and pNF: 1000, Table 1).

Table 1 also shows the results of applying a widely used imputation method^78^ to the original datasets before applying the three clustering methods. It can be seen that imputation improves the performances of all methods on (almost) all clusters, but transfer learning still outperforms clustering on the target dataset alone and simultaneous clustering on the combined dataset in some areas. Specifically, for the identification of mNP and mNFa clusters transfer learning improves the results and yields almost twice as many correct results than TargetCluster (919/1000 vs. 479/1000). TransferCluster is still the only method that identifies all three clusters in the majority of cases (919/1000, 944/1000 and 831/1000 for the three clusters of interest). In comparison, TargetCluster does not perform as well when looking at the mNP/mNFa clusters (479/1000) and ConcatenateCluster does not do as well considering the pPEP and the pNF clusters (431/1000 and 579/1000). Please note that imputation trough MAGIC^78^ greatly increased the overlap in genes between the two datasets after gene filtering from 4402 to 20125 common genes. The larger common feature space provides an explanation for the positive effect of MAGIC on the performance of clustering after concatenation or transfer learning. However, ConcatenateCluster - which also profits from the increased number of common genes - does not perform as well as TransferCluster (looking at the pPEP/pNF clusters). Hence, knowledge transfer is necessary and improves clustering regardless of whether MAGIC is used or not.

In conclusion, we show that TransferCluster is able to improve the reliability of clustering small datasets through the transfer of knowledge from a larger, biologically relevant, yet independent, dataset and that this method is improved by and amenable to existing pre-processing approaches. See Supplementary Section 4.4.2. for a detailed description of the stability analysis using source labels that were generated via NMF clustering and using level 1, 2 and 3 labels of the original publication.

## Discussion

To address challenges in the field of clustering scRNA-Seq datasets a number of methods have been presented in the literature to make use of datasets from different studies, laboratories or points in time. These approaches can be classified into two groups:

1. Multitask learning approaches that solve clustering problems of multiple datasets simultaneously while correcting for batch effects^51–63^ and
2. Transfer learning approaches that use large reference datasets to improve the clustering of target datasets that are often smaller in sample size^67–72^.

The main point of interest of this work lies in transferring knowledge without having to combine datasets and thus our focus should be on methods that fall into the second category. Rather than limiting a clustering method to a reference set of cell types^67–70^, we aim to enable the annotation of new target clusters. This leaves us with only one method, called SAVER-X^71^, that is most closely related to the present research in aiming to adjust a target dataset with information from a source dataset. By training a deep autoencoder on the target dataset and initializing it with weights obtained from training on the source dataset SAVER-X achieves denoising of the target dataset. Denoising, however, is not the only goal of our method which can additionally be used to induce certain specific properties of the source dataset into the target dataset by making use of pre-existing source labels. In contrast to our method SAVER-X also depends on large sample sizes and is not convex.

Another relevant deep-learning based approach^72^ focuses on improving the clustering of a target dataset with the help of a source dataset that does not share any cell types with the target dataset. The method is not comparable to our transfer learning approach, because we concentrate on problems where source and target data share a significant number of cell types.

For the aforementioned reasons and to our knowledge, this work presents a novel approach to a unique problem setting, that has so far not been addressed in previous literature.

Our approach can be extended to explore a number of different research directions since it is relatively easy to apply, modify and adjust. Other downstream analysis methods instead of the SC3 clustering methods or even instead of clustering in general could be used. As mentioned before it is also possible to use the proposed method to transfer knowledge from small to large datasets. Additionally, the transfer learning approach can be applied to other areas of scientific research in biological and medical fields.

Potential future research directions making adjustments to the method itself might include the incorporation of different source and target feature spaces. If *X*_*src*_ and *X*_*trg*_ only share a small set of transcripts, a loss of (probably) vital information is inevitable since only the set of genes present in both datasets can be used. Most of the multitask learning methods listed above only use the intersection of genes of all datasets when combining the datasets. Future work should thus focus on making adjustments to the method that allow the inclusion of different sets of source and target genes. One important technical point here is that scRNA-Seq experiments often make a trade-off between high cell numbers and high gene numbers. While technologies like 10X^8^ enable high cell numbers but low gene coverage, other tools like SMARTSeq2^81^ use low cell numbers, but generate high gene coverage. Ideally one would like to use a 10X dataset (or similar) to aid the clustering of a SMARTSeq2 dataset (or similar), but somehow retain the detailed gene information. In this kind of setting where the target dataset has substantially more genes than the source dataset, a simple modification of our method is straightforward: While the transfer learning procedure can be applied without changes to the genes in both source and target, all other genes in the target dataset can be left constant. A more sophisticated way to modify the method accordingly would be to make use of a learned covariance matrix over the target genes to adjust those genes that are in the target but not in the source. The same procedure can be applied in a setting where there are source genes that are not part of the target dataset.

To summarize, we propose a novel and powerful method for transferring knowledge from a well-annotated source dataset to a target dataset of smaller sample size for which new cluster annotations are desired. Source clustering labels can be incorporated as part of this knowledge when available, but are not required. The knowledge transfer procedure is based on the application of an NMF step on the source dataset before transferring the learned knowledge to the target dataset by reconstruction of a new target dataset. Finally, this modified target dataset can potentially be provided to any clustering algorithm. We have shown here that it can be successfully applied to SC3 clustering and improves the results of SC3 consistently for a range of different settings. Specifically, transferring knowledge from a large well-annotated source dataset to a smaller target dataset was not only more successful than applying SC3 to the original target set alone but also to a simple concatenation of the source and target. This was found to be true in both simulated and real-world environments where source and target were either sampled from identical distributions of cells or only shared a subset of cell clusters. In real-world applications the method will thus be especially helpful when the overlap between source and target data is not perfect and concatenation of the two datasets is not a good option. The method was shown to perform well regardless of whether reliable clustering labels of the source data are available or not. The performance of the proposed method can be further improved by applying appropriate pre-processing batch effect removal or imputation before clustering.

## Supplementary information

Supplementary  Information

## Data Availability

The datasets analyzed during the current study are available in the following GEO repositories: • Tasic *et al*. (2016)^26^: https://www.ncbi.nlm.nih.gov/geo/query/acc.cgi?acc=GSE71585. • Usoskin *et al*. (2014)^20^: https://www.ncbi.nlm.nih.gov/geo/query/acc.cgi?acc=GSE59739 • Hockley *et al*. (2018)^21^: https://www.ncbi.nlm.nih.gov/geo/query/acc.cgi?acc=GSE102962 The code for simulating scRNA-Seq datasets is available at https://github.com/nicococo/scRNA.
