# Supplementary material for: Using transfer learning from prior reference knowledge to improve the clustering of single-cell RNA-Seq data
Source: Sci Rep. 2019 Dec 30;9:20353. doi: 10.1038/s41598-019-56911-z (PMC6937257; doi:10.1038/s41598-019-56911-z)
Supplement: Supplementary file 1 — Supplementary Information [file 41598_2019_56911_MOESM1_ESM.pdf]

Supplementary Material

Using transfer learning from prior reference knowledge to improve the clustering of single-cell RNA-Seq data

Bettina Mieth, James R.F. Hockley, Nico Görnitz, Marina M.-C. Vidovic, Klaus-Robert Müller, Alex Gutteridge & Daniel Ziemek

Content of Supplementary Material

1. Supplementary methods
    1.1. SC3: Consensus clustering of single-cell RNA-seq data
    1.2. Baseline methods
2. Simulation study on generated single-cell RNA-Seq data
    2.1. Pre-processing
    2.2. Parameter selection
    2.3. Distribution of cell counts
    2.4. Results and mixture parameter selection via KTA scores
3. Analysis of Tasic data
    3.1. Pre-processing
    3.2. Parameter selection
    3.3. Ground truth cluster memberships
4. Analysis of Hockley and Usoskin data
    4.1. Pre-processing
    4.2. Parameter selection
    4.3. Source cluster memberships
    4.4. Results
        4.4.1. Results for all source cluster memberships
        4.4.2. Stability analysis
References of Supplementary Material

1. Supplementary Methods

Given a well-known source dataset with clustering labels, the proposed method improves the clustering of an unlabeled target dataset by transferring knowledge from source to target data via Non-Negative Matrix Factorization (NMF)[1,2]. The modified target dataset can then be provided to any kind of clustering algorithm. In this work we are using SC3[3] as an example. The following sections describe the exemplary clustering algorithm, SC3, in more detail and present a visualization of the baseline methods that the transfer learning approach is compared to.

1.1. SC3: Consensus clustering of single-cell RNA-seq data

SC3[3] is a well-known unsupervised clustering algorithm for scRNA-Seq data. The basic steps of the SC3 algorithm are shown in **Supplementary Figure S1**. Given an expression matrix, SC3 first applies a gene filter and log-transforms the data. Then, three cell distance matrices are calculated using Euclidean, Pearson and Spearman metrics, respectively. The three distance matrices are transformed by applying both PCA and Laplacian graph Eigen decomposition. Subsequently, k-means clustering is performed on the first *d* eigenvectors of the resulting six matrices where *d* comes from a predefined range of values. The clustering results are now combined by applying the Cluster-based Similarity Partitioning Algorithm (CSPA)[4] to compute a consensus matrix. Hierarchical clustering is finally used to cluster the resulting matrix into *k* clusters.

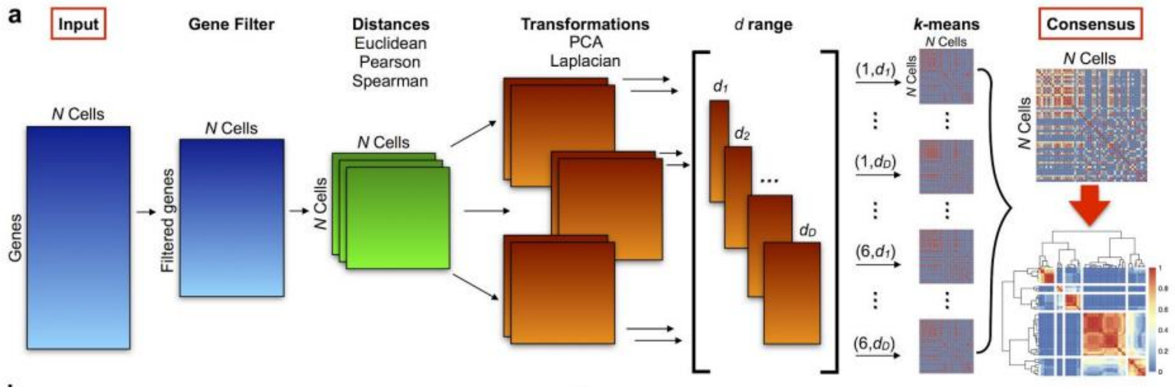

Supplementary Figure S1: The SC3 framework for consensus clustering (Kiselev 2017).

1.2. Baseline methods

For assessing the quality of our unsupervised transfer learning solution, we are interested in investigating the clustering accuracy of our method on a target dataset compared to two competitor methods. As baseline methods we implement the original SC3 clustering method on the target dataset alone (TargetCluster) and on the concatenated dataset of source and target (ConcatenateCluster). For a detailed description and a visualization of the baseline methods see **Supplementary Figure S2**.

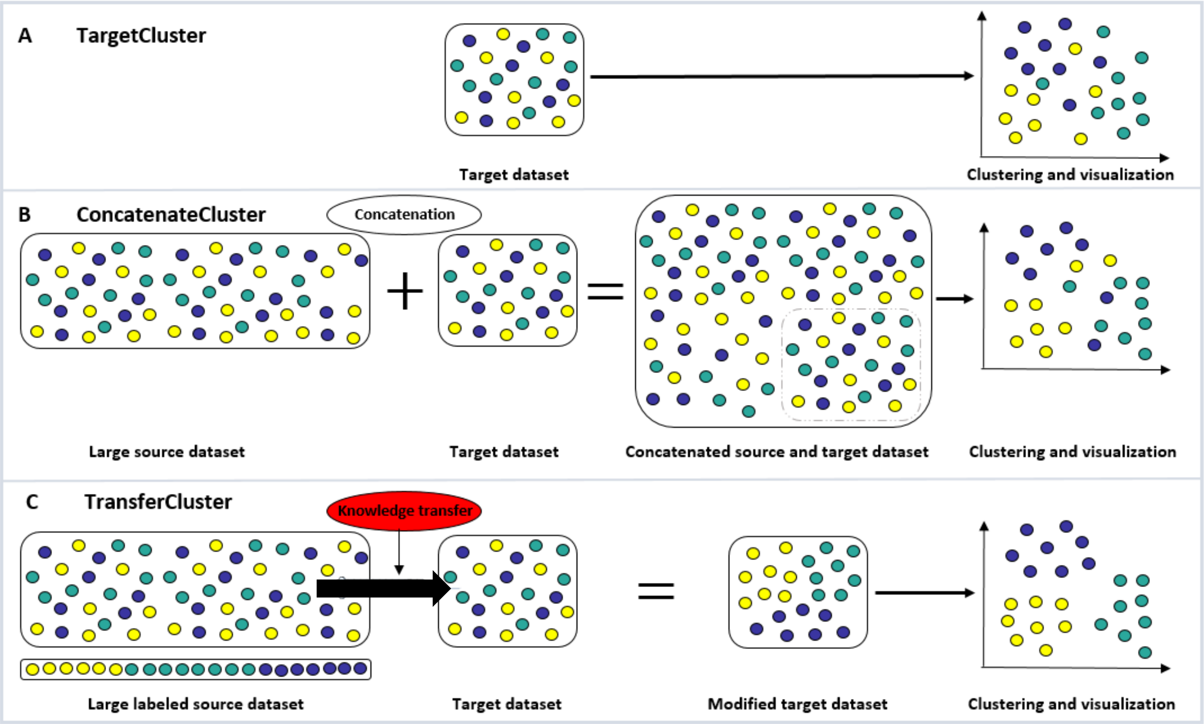

Supplementary Figure S2: Visualization of the three competitor methods of the paper. **A** TargetCluster. Clustering is applied to the target dataset alone. **B** ConcatenateCluster. Source dataset and target dataset are combined into one large dataset via simple concatenation before clustering the new dataset as a whole. Performance measures (i.e. accuracy) is calculated on the target dataset only, since it is the main focus of interest for clustering. **C** TransferCluster. The proposed method of knowledge transfer is applied to the target dataset learning from a large labeled source dataset. The resulting, modified target dataset is then provided to the clustering procedure.

2. Simulation study on generated single-cell RNA-Seq data

2.1. Pre-processing

The pre-processing steps were not applied to the generated datasets, because the generation process did not produce any unfavourable genes or cells.

2.2. Parameter selection

For each overlap setting (described in the **Method Section** of the main text) 100 datasets of 1000 source cells and 800 target cells were generated. The datasets consisted of simulated count data of 10.000 genes. All three competitor methods were applied to down sampled target datasets where for each repetition 10, 25, 50, 100, 200, 400, 600 and 800 were randomly selected from the complete target dataset.

The generated datasets where used to determine performance changes induced by varying the free parameters of the method and identify optimal settings which were assumed to be good choices for the application of the proposed method to real datasets. Here, we present the chosen values of the free parameters of the TransferCluster method which were mostly used for the investigation of the Tasic[5] and Hockley[6] data. Please refer to **Supplementary Section 3.2.** and **4.2.** for more details on the parameter selection for those datasets.

There are a number of parameters in the NMF step of the method that need specification. In the controlled environment of the generated datasets the elastic net parameters were chosen to be $\alpha = 10.0$ and $\lambda = 0.75$ and the maximum number of iterations until convergence up to a relative error of 0.001 was set to 4000. The range of mixture parameters θ to be put in the KTA score selection process was [0.0, 0.1, 0.2, 0.3, 0.4, 0.5, 0.6, 0.7, 0.8, 0.9, 1.0].

2.3. Distribution of cell counts

After generating 100 datasets with 1.800 cells and 10.000 genes the overall number of reads for each cell was counted. Each cell has a median count of 215,500 reads. The corresponding histogram is shown in **Supplementary Figure S3**.

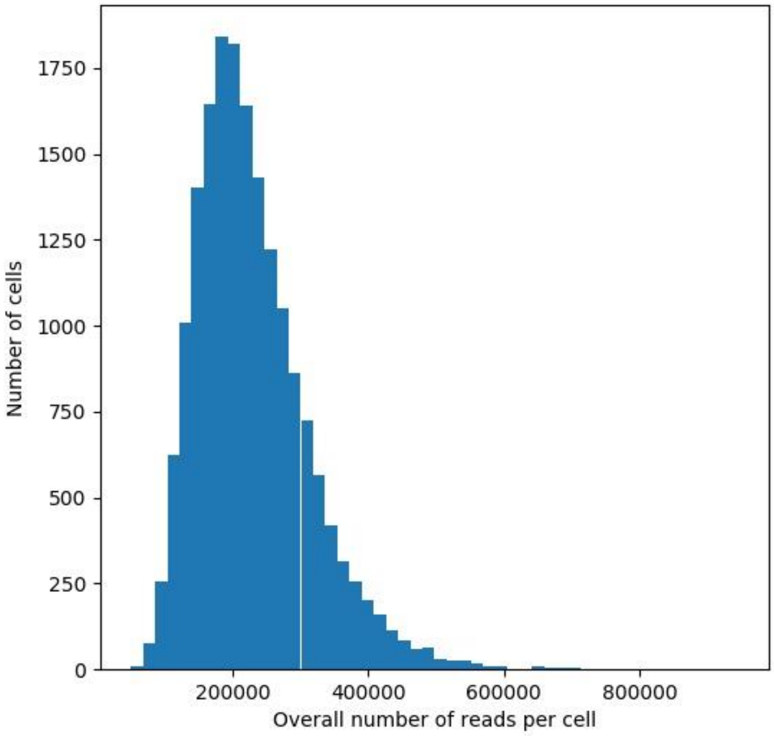

Supplementary Figure S3: Histogram of cell counts of generated datasets. 100 datasets with 1.800 cells and 10.000 genes were generated and the overall number of reads for each cell was counted.

2.4. Results and mixture parameter selection via KTA scores

The mixture parameter θ dictates how much the newly constructed target dataset should be influenced by the information of the source dataset. See the **Methods Section** of the main text for a detailed description of the parameter selection procedure of θ. It is automatically chosen via an unsupervised assessment of the clustering quality through Kernel Target Alignment (KTA) scores[7] which measure the similarity of kernels. The whole transfer learning and clustering procedure (steps 1 – 4 in the **Methods Section** of the main text) is applied with a number of values for θ within a pre-specified range and the KTA scores between the linear kernel of the mixed dataset X_{trg}^{new} (not its original version X_{trg}) over the cells and the linear kernel of the predicted labels are calculated. The scores give an indication on how well the predicted labels are represented in the mixed dataset and thus show how well the clustering procedure performs for the corresponding parameter value. The parameter value yielding the optimal KTA score is chosen as the parameter for the final clustering computation and can give an indication on the transferability between source and target data. Low values mean

source and target do not match very well (i.e. low transferability) and high values hint at high similarities (i.e. high transferability).

The simulation study on generated scRNA-Seq data was used to investigate the performance of this parameter selection procedure.

Supplementary Figure S4 gives insight into the procedure within TransferCluster that automatically selects the mixture parameter θ based on KTA scores. The first row of performance plots shows the original results on the generated datasets which can also be found in the main text of the article.
The second row presents the results of TransferCluster for a number of fixed mixture parameter values θ. The investigation of the mixture parameter θ of the proposed method for various levels of overlapping cluster structures in source and target data showed that it has to be chosen carefully. Zero mixture corresponds to not modifying the target dataset at all, i.e. not transferring any knowledge from the source dataset (equals TargetCluster). Depending on the overlap in the clustering structures of source and target data, increasing the mixture parameter might improve the performance up to a certain point and then decrease when there is an incomplete overlap. A high overlap makes the use of high mixture values necessary. If there is low or no overlap, one needs to use low values or avoid using the method.

The third row shows how the mixture parameter θ influences both the supervised performance measure ARI and its unsupervised counterpart, the KTA score, for an exemplary target sample size of 100 cells (other sample sizes show similar results). For each overlap setting, we investigate how changing the mixture parameter influences performance measured via supervised (ARI) and unsupervised accuracy measures (linear KTA). It can be seen, that the curves of the two metrics have very similar shapes for all three overlap settings and most importantly have maxima at the same or at least very close parameter values of θ. This supports the theory that KTA scores are good choice for selecting the mixture parameter θ based on the arguments of the maxima of KTA scores.

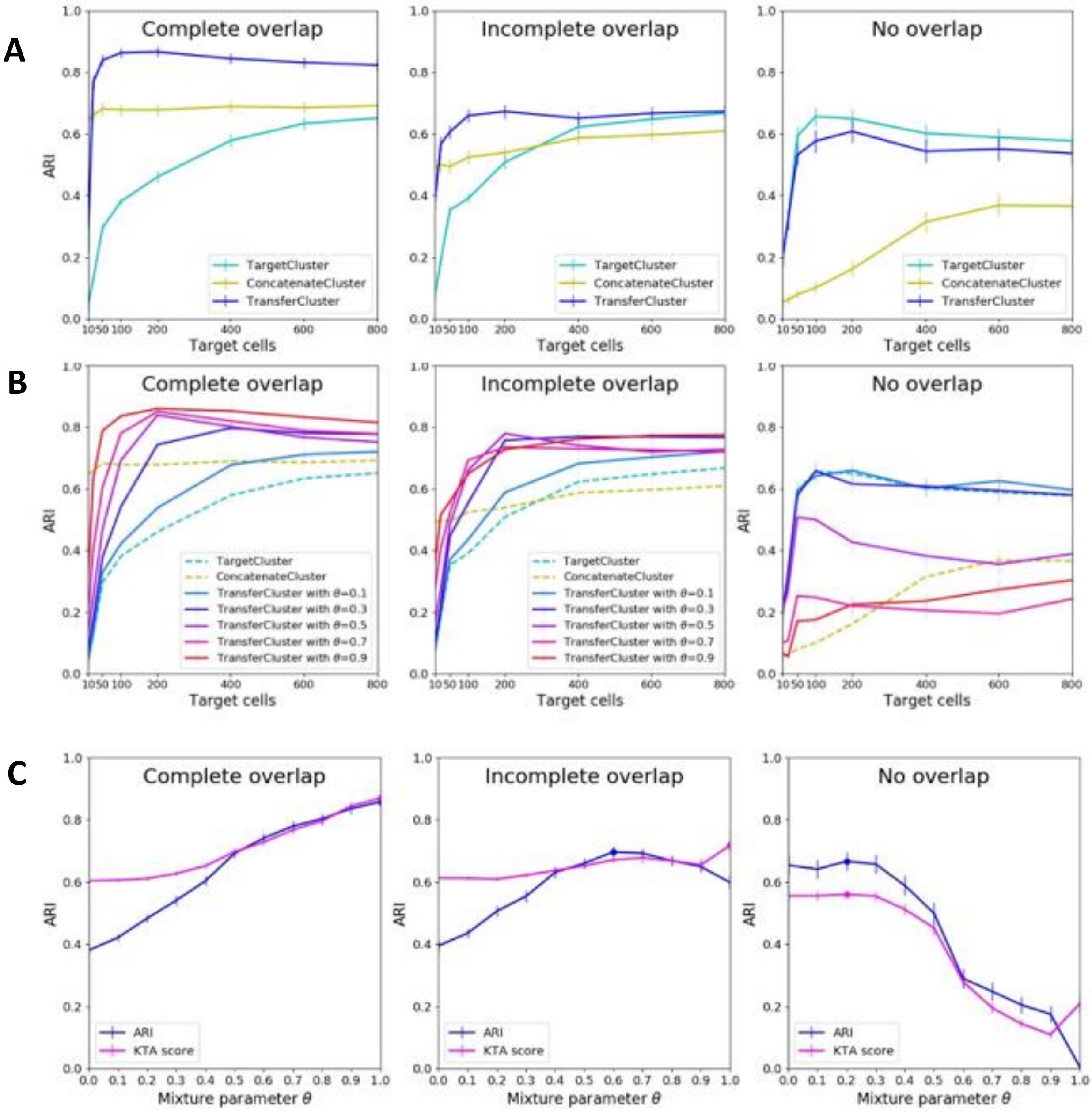

Supplementary Figure S4: Simulation study results of the transfer learning method on generated datasets and investigation of the mixture parameter selection process. **A** Main results of the three competitor methods (as seen in **Figure 2** of the main text) for three different settings of overlap in the cluster structures of source and target data: Complete, incomplete and no overlap. **B** Results of the baseline methods and TransferCluster for a number of fixed mixture parameter values θ. The complete range of θ values was [0.0, 0.1, 0.2, 0.3, 0.4, 0.5, 0.6, 0.7, 0.8, 0.9, 1.0]. Not all are shown for greater clarity. **C** Influence of the mixture parameter θ on both the supervised performance measure ARI and its unsupervised counterpart, the KTA score for an exemplary target sample size of 100 cells (other sample sizes show similar results).

3. Analysis of Tasic data

3.1. Pre-processing

Before pre-processing, the original Tasic[5] dataset contained 1679 cells and 24057 genes. The parameters of the pre-processing filters described in the **Method Section** of the main text were set to x_{genes} =2000, $x_{expression}$ =2, x_{cells} = 94 after inspection of the expression histogram in **Supplementary Figure S5**. After removing 21 cells containing fewer than 2000 genes with expression > 2 and 14510 genes with expression < 2 or > 2 in at least 94% of cells, the dataset contained expression levels of 9547 genes in 1658 cells. The expression matrix was log-transformed after adding a pseudo-count of 1.

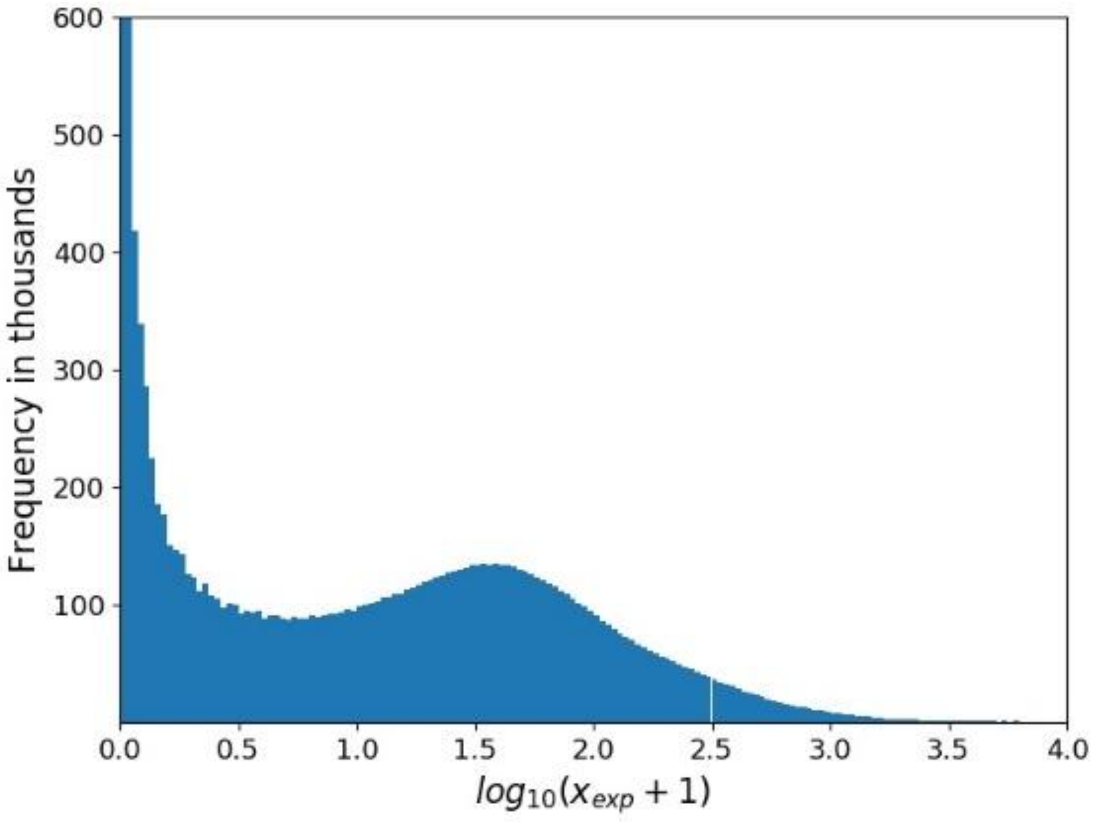

Supplementary Figure S5: Histogram of all expression values in the Tasic dataset. For 24056 genes and 1679 cells there a total of 40390024 gene expression values. 27596688 of those equal zero. x- and y-axes are cropped. The location of the frequency minimum after the zero-inflation at 0.5 implies to choose 2 as an expression cut-off value for pre-processing.

3.2. Parameter selection

The Tasic dataset was randomly split into a set of 1000 source cells and 650 target cells 100 times. The methods were applied to down sampled target datasets where for each repetition 25, 50, 100, 200, 400 and 650 were randomly selected from the complete target dataset. The number of clusters to be found by the different clustering methods was k = 18, which was the number of cell types identified in the original publication.

The free parameters in the NMF step of the method were chosen according to the best results in the controlled environment of the generated datasets, i.e. $\alpha = 10.0$ and $\lambda = 0.75$ and the maximum number of iterations until convergence up to a relative error of 0.001 was set to 4000. The range of mixture parameters θ to be put in the KTA score selection process was [0.0, 0.1, 0.2, 0.3, 0.4, 0.5, 0.6, 0.7, 0.8, 0.9, 1.0].

A number of adjustments had to be made when the data-driven clustering labels of the original publication were used for the source data (**Supplementary Table S2**) and not the generated NMF labels. After careful investigation of the Tasic data with the labels from the original publication (main text **Figure C** and **D**) it was proven to be best to avoid having very high mixture parameters. Consequently, the range of mixture parameters θ to be put in the KTA score selection process was [0.1, 0.2, 0.3, 0.4, 0.5, 0.6, 0.7]. The parameters of the NMF were set to $\alpha = 1.0$ and $\lambda = 1.0$ in this case, indicating that a strong L1 regularization is favourable.

3.3. Ground truth cluster memberships

The transfer learning approach and its baselines were investigated under two different conditions. Firstly, we assumed that no ground truth labels were available and generated labels for 18 cell clusters via NMF clustering[1,2] on the whole dataset (**Supplementary Table S1**). As it is based on the totality of the data we interpret this clustering as a ground truth clustering and apply our method and the baseline algorithms to a subset of the dataset, to see how each method performs relative to this definition of ground truth when not all of the data is available. For the source dataset those labels are put into the TransferCluster procedure. For the validation of all methods the target labels are used for measuring accuracy.

Supplementary Table S1: Ground truth cluster memberships for Tasic data generated via NMF clustering

**Cell type**	**1**	**2**	**3**	**4**	**5**	**6**	**7**	**8**	**9**	**10**	**11**	**12**	**13**	**14**	**15**	**16**	**17**	**18**
**Counts**	67	34	193	42	225	41	129	39	32	125	79	249	107	33	66	33	143	21

Secondly, we use the data-driven clustering labels provided in the original paper and take those as the ground truth labels. Specifically, we use a cut-off point in the provided clustering hierarchy that results in 18 clusters (**Supplementary Table S2**). Given those alternative ground truth labels, we once again run TargetCluster, ConcatenateCluster and TransferCluster on the Tasic data.

Supplementary Table S2: Ground truth cluster memberships for Tasic data from original publication

	**Non-neuronal cells**			**Glutamatergic cells**									**GABAergic cells**					
**Cell type**	Endothelial	Glia	SMC	L2	L2/3	L4	L5	L5a	L5b	L6	L6a	L6b	Ndnf	Igtp	Pvalb	Sncg	Sst	Vip
**Counts**	14	125	15	21	95	275	24	119	59	33	103	35	79	10	275	9	202	186

4. Analysis of Hockley and Usoskin data

4.1. Pre-processing

Before pre-processing, the original Hockley[6] dataset contained 314 cells and 45513 genes. The parameters of the pre-processing filters described in the **Method Section** of the main text were set to x_{genes} =2000, $x_{expression}$ =1, x_{cells} = 94 after inspection of the expression histogram in **Supplementary Figure S6**. No cells contained fewer than 2000 genes with expression > 1 and 35862 genes with expression < 1 or > 1 in at least 94% of cells were removed. The dataset now contained expression levels of 9651 genes in 314 cells. The expression matrix was log-transformed after adding a pseudo-count of 1.

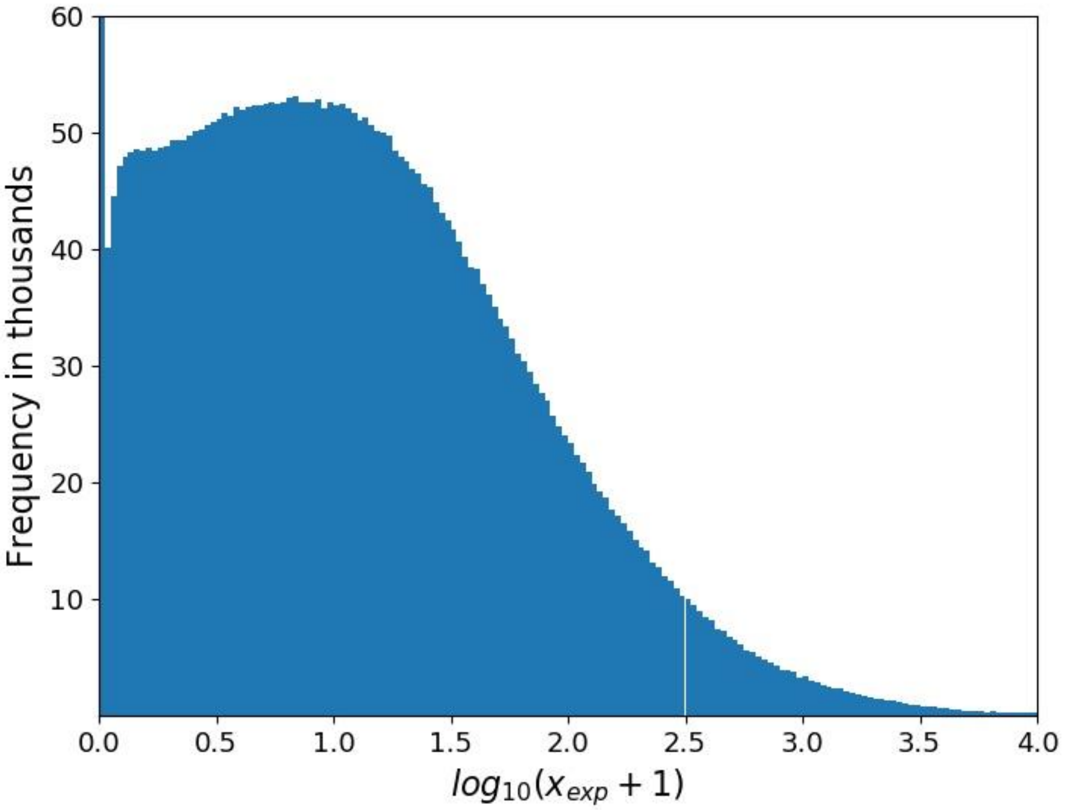

Supplementary Figure S6: Histogram of all expression values in the Hockley dataset. For 45513 genes and 314 cells there a total of 14291082 gene expression values. 10181090 of those equal zero. x- and y-axes are cropped. The location of the frequency minimum after the zero-inflation at 0.25 implies to choose 1 as an expression cut-off value for pre-processing.

Before pre-processing, the original Usoskin[8] dataset contained 622 cells and 20191 genes. The parameters of the pre-processing filters described in the **Method Section** of the main text were set to x_{genes} =2000, $x_{expression}$ =1, x_{cells} = 94 after inspection of the expression histogram in **Supplementary Figure S7**. After removing 121 cells that contained fewer than 2000 genes with expression > 1 and 10911 genes with expression < 1 or > 1 in at least 94% of cells, the dataset now contained expression levels of 9280 genes in 501 cells. The expression matrix was log-transformed after adding a pseudo-count of 1.

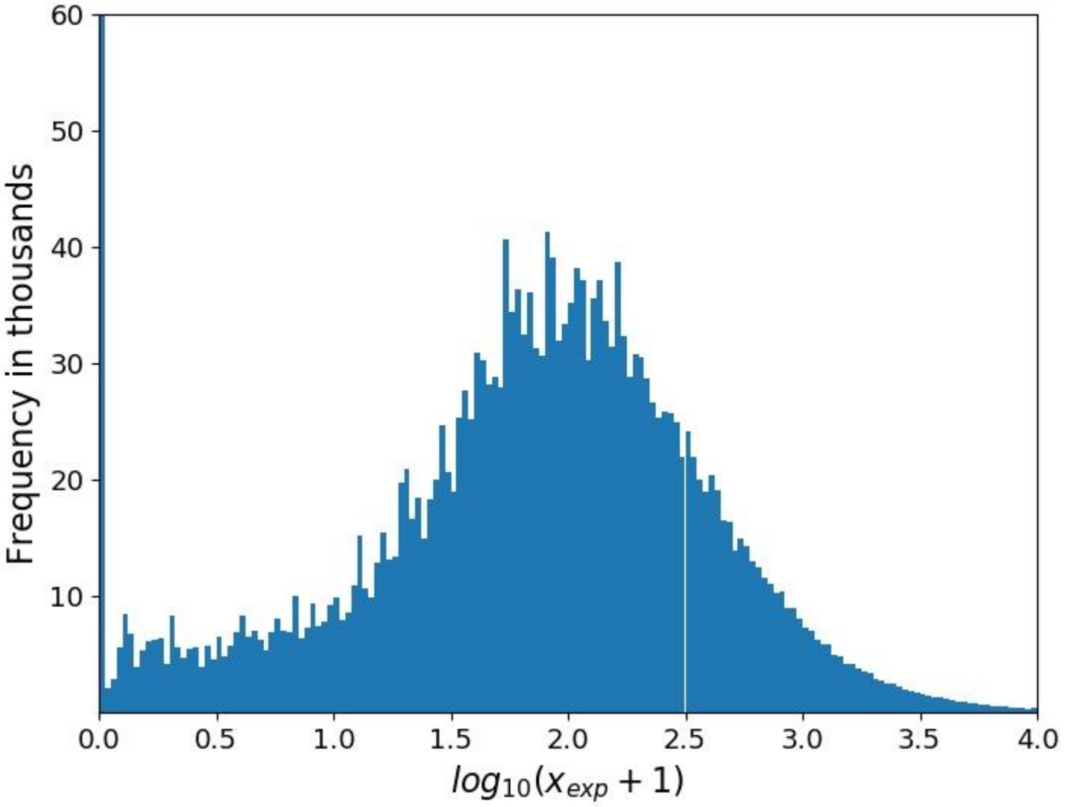

Supplementary Figure S7: Histogram of all expression values in the Usoskin dataset. For 20191 genes and 622 cells there a total of 12558802 gene expression values. 10368845 of those equal zero. x- and y-axes are cropped. The location of the frequency minimum after the zero-inflation at 0.25 implies to choose 1 as an expression cut-off value for pre-processing.

Both ConcatenateCluster and TransferCluster can only be applied when the set of genes in source and target are identical. Using only the subset of 4402 genes that appear in both source and target data the target dataset now contains 4402 genes and 314 cells and the source dataset contains 4402 genes and 501 cells.

4.2. Parameter selection

The free parameters in the NMF step of the method were chosen according to the best results in the controlled environment of the generated datasets, i.e. $\alpha = 10.0$ and $\lambda = 0.75$ and the maximum number of iterations until convergence up to a relative error of 0.001 was set to 4000. The number of clusters to be put into the different clustering methods was $k = 7$, which was the number of cell types identified in the original Hockley publication.

The mixture parameter θ is again selected automatically via the KTA score selection process (See **Methods Section** in the main paper). In **Supplementary Figure S8** we present the KTA scores for a range of θ between 0 (meaning no mixture, i.e. no transfer learning) and 1 (meaning full mixture) and note that high θ values are to be avoided and taking lower θ is to be preferred. The maximal KTA score is obtained for $\theta = 0.7$ which is the value that is consequently chosen by the automatic procedure. These findings indicate that the proposed transfer learning method was able to identify relatedness but also differences in the two datasets by automatically choosing a mixture parameter that lies in the middle of the range of possible values of θ. This is in accordance with the fact that the source and target datasets are completely independent, but biologically related, datasets, collected at different times and places.

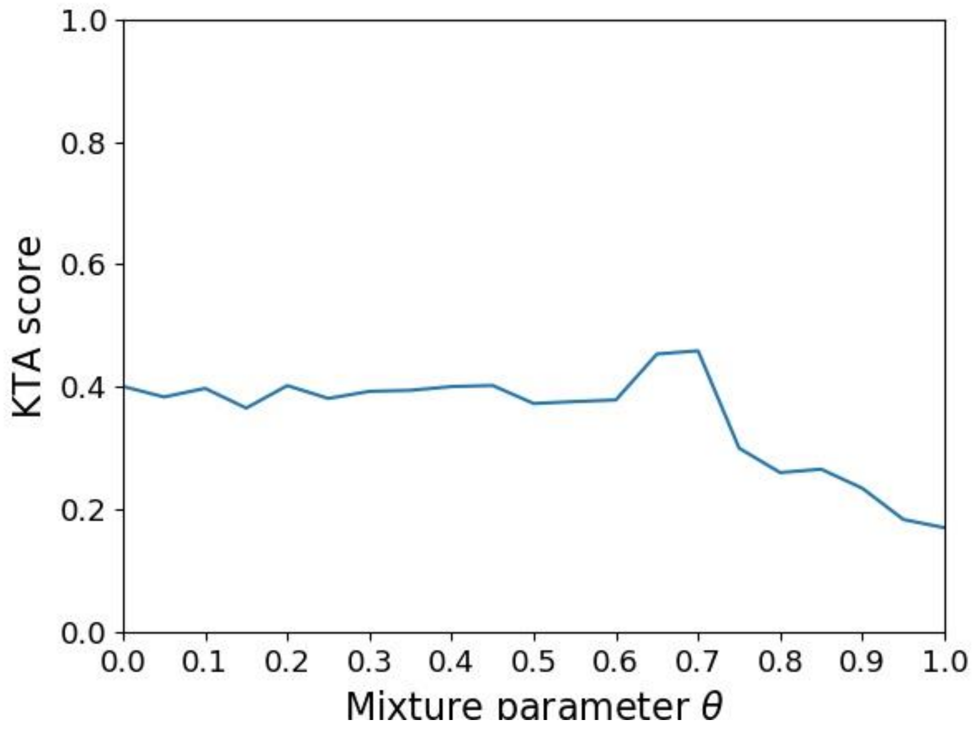

Supplementary Figure S8: Influence of the mixture parameter θ on the unsupervised performance measure - the KTA score - for the Hockley target dataset and the Usoskin source dataset. The automatic mixture parameter selection process chooses the argument of the maximum of this curve, which is 0.7.

4.3. Source cluster memberships

As with the Tasic dataset we first analyzed the Hockley data pretending no reliable source labels for the Usoskin dataset were available and generated them via NMF Clustering. We assumed a complete overlap between the cell types in source and target data and chose the number of clusters to be k = 7 for the source label generation. See **Supplementary Table S3** for the corresponding cell counts of each cluster.

Supplementary Table S3: Source cluster memberships for Usoskin data generated via NMF clustering

**Cell type**	**1**	**2**	**3**	**4**	**5**	**6**	**7**
**Counts**	29	49	112	169	55	30	57

Now, we used the source labels from the data driven clustering of the original Usoskin *et al* publication[8]. They provided labels in the form of a hierarchical clustering which was cut off at three different levels resulting in three different sets of source labels with different numbers of clusters (4,8 and 11 cell types), which are shown in **Supplementary Table S4**. In the main text of the current paper level 3 labels were used for TransferCluster. Here, we additionally present the results for NMF labels and level 1 and level 2 labels.

Supplementary Table S4: Source cluster memberships for Usoskin labels form the original publication

<table>
<tr><th></th><th>NF1</th><th>NF2</th><th>NF3</th><th>NF4</th><th>NF5</th><th>NP1</th><th>NP2</th><th>NP3</th><th>PEP1</th><th>PEP2</th><th>TH</th></tr>
<tr><td>Level 1</td><td colspan="5">139</td><td colspan="3">169</td><td colspan="2">81</td><td>233</td></tr>
<tr><td>Level 2</td><td>31</td><td colspan="2">60</td><td colspan="2">48</td><td>125</td><td colspan="2">44</td><td>64</td><td>17</td><td>233</td></tr>
<tr><td>Level 3</td><td>31</td><td>48</td><td>12</td><td>22</td><td>26</td><td>125</td><td>32</td><td>12</td><td>64</td><td>17</td><td>233</td></tr>
</table>

4.4. Results

4.4.1. Results for all source cluster memberships

Supplementary Figure S9 shows the clustering results of all competitor methods on the Hockley dataset. TargetCluster uses only data from Hockley to assign clusters and ConcatenateCluster uses a concatenation of data from Hockley and Usoskin to assign clusters. TransferCluster uses the novel transfer learning approach with Hockley as target and Usoskin as source with four different sets of corresponding labels (described in **Supplementary Section 4.3.**)

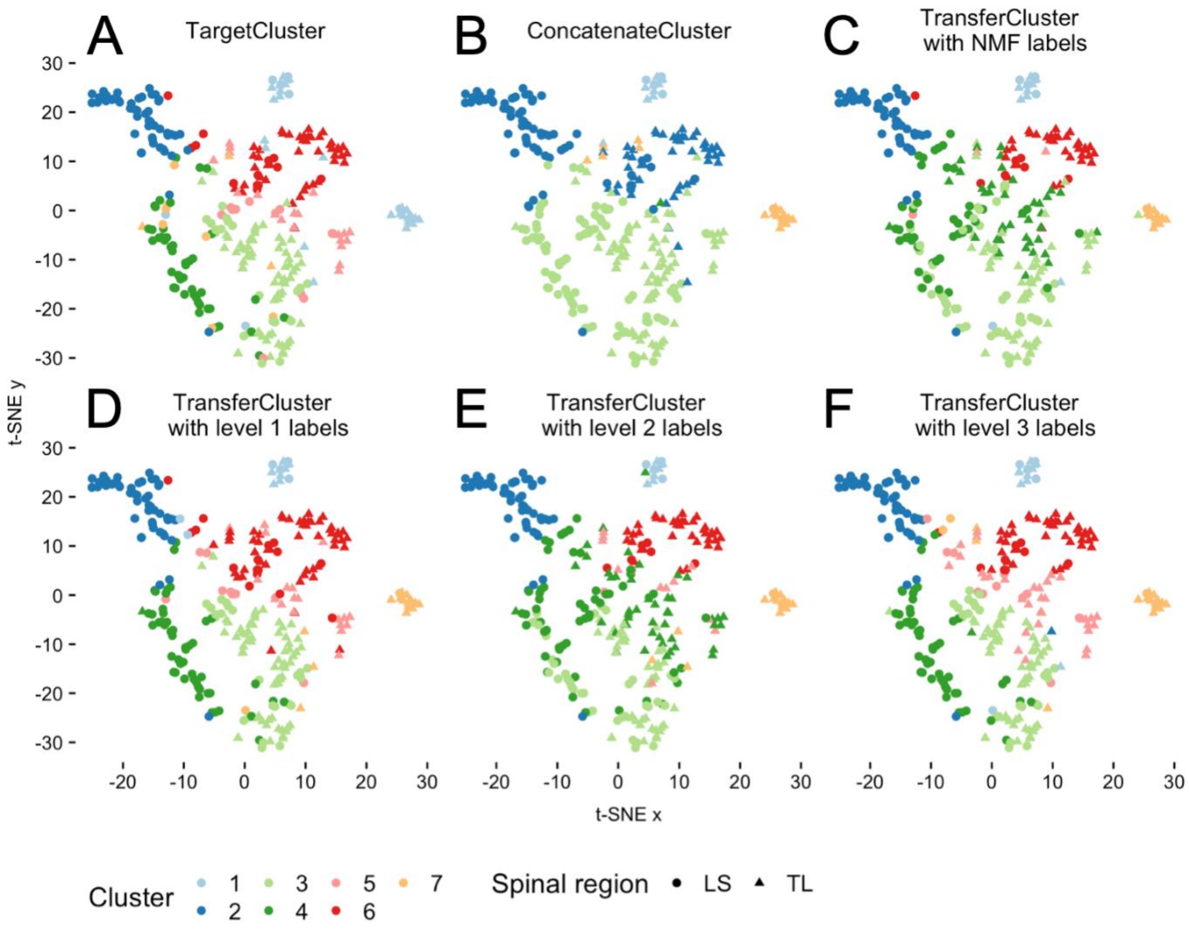

Supplementary Figure S9: t-SNE plots of the Hockley data and clustering memberships for all methods. Colour refers to clustering results of the baseline methods (TargetCluster (A) and ConcatenateCluster (B)) and the transfer learning approach with various labels of the source data (TransferCluster with NMF labels (C) and level 1 (D), 2 (E) and 3 (F) labels). Shape refers to spinal segment from which the neuron was isolated (triangle, TL (thoracolumbar); circle, LS (lumbosacral)).

4.4.2. Stability analysis

Since SC3 - the clustering method used for all approaches investigated in this paper (TargetCluster, ConcatenateCluster and TransferCluster) - is not deterministic and produces different results when solving the same clustering problem multiple times. We counted the number of times some specific clusters of interest were separated correctly from each other by the three methods when repeating the procedure 1000 times.

Three pairs of clusters were identified to be of interest and **Supplementary Table S5** shows the number of times each of those pairs of cell groups was separated correctly. Two

biologically distinct groups of cells, named mNP and mNFa cells (**Main Figure 4 G** Cluster 1 and 7), were only separated 224 times when applying SC3 on the target dataset alone. Taking source information via the proposed transfer learning method TransferCluster with NMF or level 1, 2 and 3 labels into account consistently increases this number (to 469, 300, 313 and 352, respectively). Concatenating source and target datasets and applying SC3 to the complete dataset (ConcatenateCluster) was seen to increase the number of times mNP and mNFa cells were correctly separated even further to 506. However, this came with a loss of performance when looking at the other two pairs of cell types that were only poorly separated with ConcatenateCluster. pNf cells (**Main Figure 4 G** Cluster 2 vs. 6) were only separated 481 times and the pPep cells (**Main Figure 4 G** Cluster 4 vs. 3) only 4 times. In contrast, TransferCluster was able to almost perfectly separate pNF clusters independent of what labels were used for the source data (999, 1000, 1000, 1000 for NMF, level 1, 2 and 3 labels, respectively) and also has very high separation rates for the pEP cell types (984, 703, 706 and 887 for NMF, level 1, 2 and 3 labels, respectively).

Supplementary Table S5: Stability analysis.

	**mNP/mNFa cluster separation counts**	**pNF cluster separation counts**	**pPep cluster separation counts**
**TargetCluster**	224	999	984
**ConcatenateCluster**	506	481	4
**TransferCluster with NMF labels**	469	999	984
**TransferCluster with level 1 labels**	300	1000	703
**TransferCluster with level 2 labels**	313	1000	706
**TransferCluster with level 3 labels**	352	1000	887

References of Supplementary Material

1. Lee, D.D. & Seung, H.S. Learning the parts of objects by non-negative matrix factorization. *Nature.* **401,** 788-791 (1999).
2. Lee, D.D. & Seung, H.S. Algorithms for non-negative matrix factorization. *Adv. Neural Inf. Process. Syst.* **1,** 556–562 (2001).
3. Kiselev, V. Y. *et al.* SC3: consensus clustering of single-cell RNA-seq data. *Nat. Methods.* **14,** 483–486 (2017).
4. Strehl, A. & Ghosh, J. Cluster Ensembles – A Knowledge Reuse Framework for Combining Multiple Partitions. *J. Mach. Learn. Res.* **3,** 583-617 (2002).
5. Tasic, B. *et al.* Adult mouse cortical cell taxonomy revealed by single cell transcriptomics. *Nat. Neurosci.* **19,** 335–46 (2016).
6. Hockley, J. R. F. *et al.* Single-cell RNAseq reveals seven classes of colonic sensory neuron. *Gut*. 2017–315631 (2018).
7. Cristianini, N., Shawe-Taylor, J., Elisseeff, A. & Kandola, J. On Kernel Target Alignment. *Adv. Neur. In.,* 367–373 (2002).
8. Usoskin, D. *et al.* Unbiased classification of sensory neuron types by large-scale single-cell RNA sequencing. *Nat. Neurosci.* **18,** 145–153 (2014).
